# Incidence of malignancy and related mortality after kidney transplantation: a nationwide, population-based cohort study in Korea

**DOI:** 10.1038/s41598-020-78283-5

**Published:** 2020-12-08

**Authors:** Seri Jeong, Ho Sup Lee, Seom Gim Kong, Da Jung Kim, Sangjin Lee, Min-Jeong Park, Wonkeun Song, John Hoon Rim, Hyung Jik Kim

**Affiliations:** 1 Department of Laboratory Medicine, Kangnam Sacred Heart Hospital, Hallym University College of Medicine, Seoul, 07441 South Korea; 2 Department of Hematology-Oncology, Kosin University College of Medicine, Busan, 49267 South Korea; 3 Department of Pediatrics, Kosin University College of Medicine, Busan, 49267 South Korea; 4 Department of Statistics, Graduate School, Pusan National University, Busan, 46241 South Korea; 5 Department of Pharmacology, Yonsei University College of Medicine, Seoul, 03722 South Korea; 6 Department of Medicine, Physician-Scientist Program, Yonsei University Graduate School of Medicine, Seoul, 03722 South Korea; 7 Department of Internal Medicine, Hallym University Sacred Heart Hospital, Hallym University College of Medicine, 22, Gwanpyeong-ro 170 beon-gil, Dongan-gu, 14068 Anyang-si, Gyeonggi-do South Korea

**Keywords:** Cancer epidemiology, Cancer prevention, Kidney

## Abstract

Post-transplant malignancy (PTM) is a leading cause of premature mortality among kidney transplantation recipients. However, population-based cohort studies that cover incidence, mortality, and risk factors for PTM are rarely reported, especially in East Asia. We designed a retrospective cohort study using a national population-based database. A total of 9915 kidney recipients between 2003 and 2016 were included. During this period, 598 cases (6.0%) of de novo PTM occurred. The most common PTM was thyroid cancer (14.2%), followed by colorectal (11.2%), kidney (10.7%), and stomach cancers (8.9%). The standardised incidence ratio for all-site cancer was 3.9. The risks of Kaposi sarcoma (192.9) and kidney cancer (21.1) were more than 10 times those of the general population. Cancer-related deaths were 89 (14.9%) with liver cancer being the highest (14.6%), followed by lung cancer (13.5%), non-Hodgkin lymphoma (NHL) (12.4%), stomach cancer (9.0%), and colorectal cancer (7.9%). The standardised mortality ratio (SMR) was slightly elevated (1.4). A notable increase in SMR was observed for lymphoma (9.3 for Hodgkin lymphoma and 5.5 for NHL). Older age and graft failure were significantly related to PTM. These findings reflecting geographical variation have implications for the development of strategies for fatal cancers to prevent premature deaths from PTM.

## Introduction

Post-transplant malignancy (PTM) is a devastating complication of kidney transplantation and the second most common cause of mortality in kidney recipients^1^. In general, kidney transplant recipients have a higher incidence of malignancies compared with the general population^1,2^. Poorer outcomes, including mortality and graft failure, have been reported in patients with PTM^2–5^. However, the incidence, mortality, and risk factors for PTM show considerable ethnic and geographic variation. Non-melanoma skin cancer (NMSC) and non-Hodgkin lymphoma (NHL) are the most common PTMs in the United States and Europe^6–9^. In contrast, stomach and kidney cancers are commonly reported PTMs in Asian countries^10–12^.

There have only been a few reports of PTM in South Korea. Hwang et al.^11^ presented stomach cancer and malignant lymphoma as the most common PTMs based on a 40-year single-centre dataset. Most previous studies are derived from single-centre data with a relatively small number of recipients^13–15^. Recently Heo et al.^16^ and Park et al.^17^ found thyroid cancer to be the most common PTMs identified in from 5- and 8-year nationwide databases, respectively. The relatively short mean duration of follow-up may affect the accuracy of data concerning late-onset PTM. Furthermore, mortality data derived from PTM and risk factors for PTM were limited in the previous studies. Reports from Western countries have yielded contradictory results on cancer mortality; risk of death in USA transplant recipients was not increased^18^, but those in Canada^3^, Australia, and New Zealand^19^ were three times higher than the general population. A large population-based study with a lengthy follow-up is essential in order to understand PTM and establish cancer surveillance strategies for kidney recipients.

The present study was based on the use of a comprehensive database operated by the National Health Insurance (NHI) of the Korean government. This database contains all the records of healthcare utilisation by kidney recipients who were enrolled in the Rare Intractable Disease (RID) system for reimbursement purposes. Registration in such is confirmed by a certified physician based on the RID criteria, which reflect international guidelines, and is verified by the NHI system. Therefore, the use of this database was suitable for the investigation of PTM among kidney recipients.

Using this database, we performed a comprehensive nationwide population-based analysis with the permitted maximum lengthy follow-up duration to investigate the incidence, mortality, and risk factors for PTM. This study can facilitate prevention and management of PTMs, which will contribute to outcome improvements in kidney recipients.

## Results

### Characteristics of patients

A total of 9915 patients who underwent kidney transplantation from 2003 to 2016 were included in our study cohort. The median follow-up duration was 4.87 years, representing 53,835 person-years of follow-up. During this period, 598 cases of de novo PTM occurred. The baseline characteristics of these patients are presented in Table 1. Patients with PTM were older than the non-PTM group (median age: 48 vs. 46 years, *P* < 0.001). There were significant differences between the PTM and non-PTM groups for immunosuppressive agents: induction (*P* = 0.004 for basiliximab, and *P* = 0.002 for anti-thymocyte globulin), and maintenance (*P* < 0.001 for calcineurin inhibitors, mycophenolate mofetil, and corticosteroid). There was a higher proportion of recipients who suffered from graft failure in the PTM group than the non-PTM group (*P* < 0.001). The proportion of PTM cases decreased from 383 (64.1%) in 2003–2009 to 215 (36.03%) in 2010–2016.

**Table 1:** Comparison of characteristics between patients with post-transplant malignancies (PTM) versus without PTM.

Variable*	Total patients	PTM	Non-PTM	*P*-value
**Age, years**				
< 40	3225 (32.5)	154 (25.8)	3071 (33.0)	0.001
40–59	5736 (57.9)	377 (63.0)	5359 (57.5)	
> 59	954 (9.6)	67 (11.2)	887 (9.5)	
**Sex**				
Male	5970 (60.2)	341 (57.0)	5629 (60.4)	0.110
Female	3945 (39.8)	257 (43.0)	3688 (39.6)	
**Total person-years**	53,835	2809	51,026	
Male	32,175	1619	30,556	
Female	21,660	1190	20,470	
**Induction immunosuppressant**				
Basiliximab	1135 (11.4)	47 (7.9)	1088 (11.7)	0.004
Anti-thymocyte globulin	7821 (78.9)	442 (73.9)	7379 (79.2)	0.002
Maintenance immunosuppressant				
Tacrolimus + MMF + corticosteroid	6958 (70.2)	355 (59.4)	6603 (70.9)	< 0.001
Cyclosporine + MMF + corticosteroid	2158 (21.8)	210 (35.1)	1948 (20.9)	< 0.001
**Infection**				
CMV disease	1018 (10.3)	61 (10.2)	957 (10.3)	0.956
EBV mononucleosis	11 (0.1)	1 (0.2)	10 (0.1)	0.670
Graft failure	442 (4.5)	63 (10.5)	408 (4.4)	< 0.001
**Year of transplant**				
2003–2009	3473 (35.0)	383 (64.1)	3090 (33.2)	< 0.001
2010–2016	6442 (65.0)	215 (36.0)	6227 (66.8)	

*CMV* cytomegalovirus, *EBV* Epstein–Barr virus, *MMF* mycophenolate mofetil, *PTM* post-transplant malignancy. *Data are expressed as number and percentage.

### Incidence of PTM

The overall incidence of PTM was 6.0% (598/9915). The cancer types and characteristics of PTMs are presented in Table 2. The most common PTM was thyroid cancer (n = 85, 14.2%), followed by colorectal cancer (n = 67, 11.2%), kidney cancer (n = 64, 10.7%), stomach cancer (n = 53, 8.9%), and prostate cancer (n = 49, 8.2%). The median age of patients at diagnosis of PTM was 52.0 years (interquartile range: 16.0 years). Melanoma (40.0 years), cervical cancer (44.5 years), and oral cavity cancer (47.0 years) occurred in younger recipients, who were also relatively younger at the time of kidney transplantation. In contrast, oesophageal cancer (65.0 years), pancreatic cancer (60.0 years), ureter cancer (59.5 years), and NMSC (59.0 years) developed in older recipients, resulting in relatively longer duration from transplantation to PTM. The median interval between transplantation and PTM was 4.0 years (interquartile range: 4.8 years). PTM occurred in 49 (8.2%) recipients within 1 year, 305 (51.0%) between 1 and 5 years, 198 (33.1%) between 6 and 10 years, and 46 (7.7%) over 10 years. Kaposi sarcoma, gallbladder, oral cavity, and pancreatic cancers developed within 3 years after transplantation, whereas ureter, oesophageal, and NMSC cancers occurred later post-transplant.

**Table 2:** Distribution of post-transplant malignancies.

Type of cancer	Number of cancer (%)			Age at transplant, years*			Age at diagnosis of PTM, years*			Time between transplant and PTM, years*		
	Male	Female	Total	Male	Female	Total	Male	Female	Total	Male	Female	Total
Thyroid	30 (8.8)	55 (21.4)	85 (14.2)	44.5 (38.8–54.8)	44.0 (36.0–50.0)	44.0 (37.0–52.0)	48.5 (43.0–56.8)	46.0 (41.0–53.0)	48.0 (42.0–55.0)	2.7 (1.8–6.5)	4.0 (2.1–6.6)	3.4 (1.8–6.5)
Colorectal	34 (10.0)	33 (12.8)	67 (11.2)	46.5 (39.3–56.5)	47.0 (34.0–54.0)	47.0 (36.5–55.0)	52.5 (45.3–59.0)	51.0 (38.0–59.0)	52.0 (42.0–59.0)	3.9 (2.3–8.2)	4.5 (2.2–8.5)	4.4 (2.2–8.4)
Kidney	46 (13.5)	18 (7.0)	64 (10.7)	44.0 (37.3–52.8)	45.0 (36.5–53.0)	45.0 (36.8–53.0)	51.0 (41.3–59.0)	53.0 (43.3–58.3)	51.5 (41.8–59.0)	6.1 (2.4–9.1)	3.6 (2.1–8.2)	5.8 (2.3–9.0)
Stomach	31 (9.1)	22 (8.6)	53 (8.9)	52.0 (47.0–55.5)	52.0 (46.0–58.0)	52.0 (46.0–58.0)	57.0 (49.5–62.0)	56.0 (51.3–62.0)	57.0 (51.0–62.0)	5.2 (3.3–7.0)	4.8 (2.7–6.5)	5.0 (3.3–6.8)
Prostate	49 (14.4)	0 (0.0)	49 (8.2)	52.0 (45.0–58.0)	-	52.0 (45.0–58.0)	55.0 (49.0–60.0)	-	55.0 (49.0–60.0)	3.6 (1.3–6.4)	-	3.6 (1.3–6.4)
Liver	32 (9.4)	10 (3.9)	42 (7.0)	51.0 (45.8–55.0)	49.5 (31.5–51.5)	49.5 (40.3–54.8)	54.5 (47.0–59.0)	45.5 (35.0–56.0)	53.5 (45.3–58.8)	3.3 (1.7–6.0)	2.7 (1.8–4.6)	3.2 (1.8–5.7)
Lung	31 (9.1)	11 (4.3)	42 (7.0)	54.0 (45.5–59.0)	53.5 (41.0–55.0)	53.5 (42.8–58.0)	59.0 (49.0–63.5)	53.0 (44.0–58.5)	58.0 (47.3–62.8)	4.3 (2.7–6.0)	3.8 (1.0–5.3)	4.2 (1.9–5.5)
Breast	1 (0.3)	35 (13.6)	36 (6.0)	51.0 (51.0–51.0)	44.0 (39.5–50.0)	44.0 (39.8–50.3)	57.0 (57.0–57.0)	48.0 (44.5–53.0)	48.0 (44.8–53.8)	6.3 (6.3–6.3)	4.1 (2.2–5.8)	4.2 (2.3–6.0)
Non-Hodgkin lymphoma	13 (3.8)	14 (5.4)	27 (4.5)	32.0 (29.0–47.0)	45.0 (39.8–52.0)	45.0 (31.5–51.0)	38.0 (35.0–49.0)	53.0 (41.8–57.3)	49.0 (37.0–55.0)	3.3 (1.6–9.0)	5.3 (2.4–7.1)	5.1 (1.7–7.8)
Urinary bladder	13 (3.8)	7 (2.7)	20 (3.3)	52.0 (39.0–55.0)	48.5 (41.5–51.0)	48.5 (38.8–54.3)	54.0 (48.0–57.0)	51.0 (48.0–54.5)	53.5 (47.8–57.0)	5.8 (2.5–8.7)	5.3 (2.7–6.2)	5.4 (2.4–8.1)
Cervix	0 (0.0)	14 (5.4)	14 (2.3)	-	39.5 (37.0–45.3)	39.5 (37.0–45.3)	-	44.5 (41.0–49.8)	44.5 (41.0–49.8)	-	3.8 (2.3–6.0)	3.8 (2.3–6.0)
Non-melanoma skin	9 (2.6)	4 (1.6)	13 (2.2)	54.0 (44.0–59.0)	54.0 (49.8–58.0)	54.0 (44.0–59.0)	61.0 (52.0–66.0)	56.5 (53.5–62.3)	59.0 (52.0–66.0)	7.4 (4.3–8.9)	5.1 (4.1–6.6)	7.2 (4.3–8.9)
Oral cavity	9 (2.6)	4 (1.6)	13 (2.2)	46.0 (40.0–48.0)	46.0 (36.0–41.5)	46.0 (37.0–48.0)	50.0 (41.0–53.0)	41.0 (39.3–43.5)	47.0 (41.0–51.0)	2.8 (1.9–4.9)	3.1 (1.7–4.6)	2.8 (1.8–4.9)
Pancreas	9 (2.6)	2 (0.8)	11 (1.8)	57.0 (54.0–61.0)	57.0 (37.5–54.5)	57.0 (53.5–62.0)	60.0 (56.0–61.0)	50.0 (42.0–58.0)	60.0 (54.5–63.5)	2.8 (0.9–3.5)	4.3 (3.6–4.9)	2.9 (1.0–3.6)
Leukaemia	6 (1.8)	5 (1.9)	11 (1.8)	47.0 (41.0–50.8)	47.0 (33.0–48.0)	47.0 (36.0–50.0)	50.0 (44.3–54.3)	46.0 (37.0–53.0)	48.0 (40.0–53.5)	3.7 (2.3–5.5)	4.4 (1.6–6.7)	4.2 (1.8–6.3)
Nasopharyngeal	8 (2.3)	2 (0.8)	10 (1.7)	49.5 (47.0–53.0)	49.0 (48.3–48.8)	49.0 (48.3–52.3)	53.0 (49.0–57.3)	51.0 (50.0–52.0)	52.5 (49.5–56.3)	3.5 (2.4–5.8)	3.2 (2.4–4.1)	3.5 (1.8–5.0)
Kaposi sarcoma	8 (2.3)	1 (0.4)	9 (1.5)	42.5 (33.0–55.5)	46.0 (63.0–63.0)	46.0 (34.0–57.0)	47.0 (34.0–57.0)	65.0 (65.0–65.0)	55.0 (35.0–60.0)	2.2 (1.1–5.0)	2.4 (2.4–2.4)	2.4 (1.1–4.2)
Ovarian	0 (0.0)	8 (3.1)	8 (1.3)	–	44.5 (40.0–50.8)	44.5 (40.0–50.8)	–	50.5 (43.8–53.8)	50.5 (43.8–53.8)	–	3.5 (2.9–5.2)	3.5 (2.9–5.2)
Uterus	0 (0.0)	7 (2.7)	7 (1.2)	–	52.0 (36.0–55.5)	52.0 (36.0–55.5)	–	55.0 (42.5–56.0)	55.0 (42.5–56.0)	–	4.7 (1.9–7.0)	4.7 (1.9–7.0)
Gallbladder	5 (1.5)	1 (0.4)	6 (1.0)	56.0 (51.0–57.0)	53.5 (49.0–49.0)	53.5 (49.5–56.8)	58.0 (52.0–61.0)	56.0 (56.0–56.0)	57.0 (53.0–60.3)	1.7 (1.7–3.2)	7.1 (7.1–7.1)	2.5 (1.7–5.3)
Ureter	1 (0.3)	3 (1.2)	4 (0.7)	56.0 (56.0–56.0)	52.5 (48.5–54.5)	52.5 (48.8–57.0)	61.0 (61.0–61.0)	58.0 (56.0–63.5)	59.5 (57.0–63.0)	5.3 (5.3–5.3)	9.6 (7.3–10.0)	7.5 (5.2–9.8)
Oesophagus	2 (0.6)	1 (0.4)	3 (0.5)	57.5 (55.3–59.8)	58.0 (58.0–58.0)	58.0 (55.5–60.0)	67.0 (66.0–68.0)	65.0 (65.0–65.0)	65.0 (65.0–67.0)	9.6 (8.4–10.9)	7.1 (7.1–7.1)	7.2 (7.2–9.6)
Central nervous system	2 (0.6)	0 (0.0)	2 (0.3)	53.5 (51.3–55.8)	–	53.5 (51.3–55.8)	55.5 (53.8–57.3)	–	55.5 (53.8–57.3)	2.3 (1.7–2.9)	–	2.3 (1.7–2.9)
Melanoma	1 (0.3)	0 (0.0)	1 (0.2)	36.0 (36.0–36.0)	–	36.0 (36.0–36.0)	40.0 (40.0–40.0)	–	40.0 (40.0–40.0)	4.2 (4.2–4.2)	–	4.2 (4.2–4.2)
Hodgkin lymphoma	1 (0.3)	0 (0.0)	1 (0.2)	53.0 (53.0–53.0)	–	53.0 (53.0–53.0)	53.0 (53.0–53.0)	–	53.0 (53.0–53.0)	0.8 (0.8–0.8)	–	0.8 (0.8–0.8)

*Data are expressed as median and interquartile range.

The kidney recipients had 3.9 times higher cases of development of any type of cancer when compared with the general Korean population (Table 3). The standardised incidence ratio (SIR) for PTM was higher in female than in male recipients (4.5 vs. 3.3). The SIR of Kaposi sarcoma (192.9), kidney cancer (21.1), and ureter cancer (14.5) were increased more than tenfold while those of gallbladder (1.1), central nervous system (1.2), and oesophageal (1.7) cancers were not significantly higher compared with the general population. In terms of age classes, the SIR of kidney recipients aged less than 40 years showed the highest values for PTMs (16.1 for total PTM, 6.5 for thyroid cancer, 40.0 for colorectal cancer, and 110.8 for kidney cancer) (Supplementary Table S1).

**Table 3:** Standardised incidence ratios according to the type of post-transplant malignancy.

Type of cancer	Observed rates of cancer cases			Expected rates of cancer cases*			Standardised incidence ratio (95% CI)		
	Male	Female	Total	Male	Female	Total	Male	Female	Total
Thyroid	93.2	253.9	157.9	15.7	72.9	44.2	5.9 (4.8–7.2)	3.5 (3.1–3.9)	3.6 (3.0–4.2)
Colorectal	105.7	152.4	124.5	45.4	25.2	34.2	2.3 (1.9–2.8)	6.0 (5.1–7.1)	3.6 (3.0–4.3)
Kidney	143.0	83.1	118.9	8.3	3.3	5.6	17.1 (14.4–20.1)	25.2 (20.1–31.2)	21.1 (17.4–25.2)
Stomach	96.3	101.6	98.4	62.6	26.0	42.3	1.5 (1.2–1.9)	3.9 (3.2–4.7)	2.3 (1.9–2.8)
Prostate	152.3	0.0	91.0	22.0	-	9.2	6.9 (5.9–8.1)	–	9.9 (7.9–12.1)
Liver	99.5	46.2	78.0	46.8	15.7	31.3	2.1 (1.7–2.6)	2.9 (2.1–3.9)	2.5 (2.0–3.1)
Lung	96.3	50.8	78.0	47.9	14.6	28.5	2.0 (1.6–2.4)	3.5 (2.6–4.6)	2.7 (2.2–3.4)
Breast	3.1	161.6	66.9	0.2	44.6	22.5	15.5 (3.1–43.8)	3.6 (3.1–4.2)	3.0 (2.3–3.8)
Non-Hodgkin lymphoma	40.4	64.6	50.2	7.6	5.2	6.4	5.3 (3.7–7.1)	12.3 (9.5–15.7)	7.9 (5.8–10.4)
Urinary bladder	40.4	32.3	37.2	9.2	1.7	4.9	4.4 (3.1–5.9)	19.5 (13.2–27.3)	7.6 (5.4–10.5)
Cervix	0.0	64.6	26.0	–	12.9	6.6	–	5.0 (3.9–6.4)	3.9 (2.6–5.8)
Non-melanoma skin	28.0	18.5	24.1	4.4	4.0	4.2	6.4 (4.2–9.1)	4.6 (2.6–7.1)	5.7 (3.6–8.4)
Oral cavity	28.0	18.5	24.1	4.3	1.7	2.9	6.6 (4.3–9.3)	10.7 (6.2–16.4)	8.3 (5.3–12.3)
Pancreas	28.0	9.2	20.4	8.4	5.1	6.6	3.3 (2.2–4.8)	1.8 (0.8–3.3)	3.1 (1.9–4.7)
Leukaemia	18.6	23.1	20.4	5.7	4.1	4.8	3.3 (1.9–5.1)	5.6 (3.6–8.4)	4.3 (2.5–6.4)
Nasopharyngeal	24.9	9.2	18.6	6.5	1.0	3.4	3.8 (2.4–5.6)	9.6 (4.3–17.8)	5.4 (3.2–8.5)
Kaposi sarcoma	24.9	4.6	16.7	0.1	0.0	0.1	219.4 (139.2–320.4)	–	192.9 (109.9–306.9)
Ovarian	0.0	36.9	14.9	–	6.3	3.2	–	5.9 (4.1–8.1)	4.6 (2.5–7.5)
Uterus	0.0	32.3	13.0	–	5.7	2.9	–	5.7 (3.8–7.9)	4.5 (2.4–7.7)
Gallbladder	15.5	4.6	11.1	10.1	9.8	9.9	1.5 (0.9–2.5)	0.5 (0.1–1.1)	1.1 (0.6–2.0)
Ureter	3.1	13.9	7.4	0.7	0.3	0.5	4.2 (0.8–11.7)	44.2 (23.3–73.0)	14.5 (5.5–28.1)
Oesophagus	6.2	4.6	5.6	6.7	0.5	3.2	0.9 (0.3–2.0)	9.6 (2.8–22.8)	1.7 (0.6–3.9)
Central nervous system	6.2	0.0	3.7	3.5	2.8	3.2	1.8 (0.6–3.7)	–	1.2 (0.3–3.0)
Melanoma	3.1	0.0	1.9	0.7	0.7	0.7	4.2 (0.8–12.0)	–	2.8 (0.2–9.5)
Hodgkin lymphoma	3.1	0.0	1.9	0.6	0.3	0.4	5.6 (1.1–15.8)	–	4.4 (0.3–15.3)
Total	1059.8	1186.5	1110.8	322.1	265.4	283.1	3.3 (3.1–3.5)	4.5 (4.2–4.7)	3.9 (3.7–4.2)

*CI* confidence interval. *The expected rates of cancer cases are based on the general population in Korea, adjusted for age and sex.

### Mortality of PTM

Of the 598 recipients with PTM, 89 (14.9%; 55 males and 34 females) died during the study period. The most common cause of cancer-related death in these patients was liver cancer (n = 13, 14.6%), followed by lung cancer (n = 12, 13.5%), NHL (n = 11, 12.4%), stomach cancer (n = 8, 9.0%), and colorectal cancer (n = 7, 7.9%). The standardised mortality ratio (SMR) of total recipients with PTM was 1.4 and that of female recipients (1.8) was significantly higher than that of male recipients (1.1) (Table 4). Patients with lymphoma (9.3 for Hodgkin lymphoma and 5.5 for NHL), NMSC (7.0), kidney cancer (5.8), and Kaposi sarcoma (5.6) showed significantly higher SMRs than the general population.

**Table 4:** Standardised mortality ratios according to the types of post-transplant malignancies.

Type of cancer	Observed rates of cancer death			Expected rates of cancer death*			Standardised mortality ratio (95% CI)		
	Male	Female	Total	Male	Female	Total	Male	Female	Total
Liver	31.1	13.9	24.1	35.0	11.4	23.1	0.9 (0.6–1.3)	1.2 (0.6–2.0)	1.0 (0.7–1.5)
Lung	31.1	9.2	22.3	47.5	17.0	32.3	0.7 (0.4–0.9)	0.5 (0.2–1.0)	0.7 (0.4–1.0)
Non-Hodgkin lymphoma	15.5	27.7	20.4	4.1	3.2	3.7	3.8 (2.1–6.2)	8.7 (5.7–12.5)	5.5 (3.3–8.3)
Stomach	21.8	4.6	14.9	26.5	14.3	20.4	0.8 (0.5–1.2)	0.3 (0.1–0.8)	0.7 (0.4–1.2)
Colorectal	9.3	18.5	13.0	5.6	4.3	4.9	1.7 (0.7–3.1)	4.3 (2.5–6.6)	2.7 (1.4–4.5)
Kidney	6.2	13.9	9.3	2.2	0.9	1.6	2.8 (1.0–5.9)	15.4 (8.1–25.4)	5.8 (2.6–10.7)
Nasopharyngeal	12.4	0.0	7.4	3.1	0.9	2.0	4.0 (2.0–6.8)		3.7 (1.4–7.2)
Urinary bladder	6.2	4.6	5.6	3.3	1.1	2.2	1.9 (0.7–4.0)	4.2 (1.2–10.0)	2.5 (0.9–5.6)
Ovarian	0.0	13.9	5.6	0.0	3.5	1.7		4.0 (2.1–6.5)	3.3 (1.1–7.3)
Prostate	9.3	0.0	5.6	5.0	0.0	2.5	1.9 (0.8–3.4)		2.2 (0.8–4.9)
Non-melanoma skin	3.1	9.2	5.6	0.8	0.8	0.8	3.9 (0.8–11.0)	11.5 (5.1–21.4)	7.0 (2.4–15.5)
Breast	0.0	13.9	5.6	0.1	7.5	3.8	0.0	1.8 (1.0–3.0)	1.5 (0.5–3.3)
Kaposi sarcoma	6.2	4.6	5.6	1.0	0.9	1.0	6.2 (2.2–13.1)	5.1 (1.5–12.2)	5.6 (1.9–12.4)
Pancreas	6.2	0.0	3.7	9.0	8.0	8.0	0.7 (0.2–1.5)		0.5 (0.1–1.2)
Central nervous system	6.2	0.0	3.7	2.6	2.2	2.4	2.4 (0.8–5.0)		1.5 (0.4–4.0)
Cervix	0.0	9.2	3.7	0.0	4.0	2.0		2.3 (1.0–4.3)	1.9 (0.4–4.8)
Thyroid	0.0	4.6	1.9	0.4	1.0	0.7		4.6 (1.4–11.0)	2.7 (0.2–9.2)
Leukaemia	0.0	4.6	1.9	4.1	3.1	3.6		1.5 (0.4–3.5)	0.5 (0.0–1.8)
Oesophagus	3.1	0.0	1.9	5.4	0.5	3.0	0.6 (0.1–1.6)		0.6 (0.0–2.1)
Ureter	0.0	4.6	1.9	0.7	0.5	0.6		9.2 (2.7–21.9)	3.1 (0.2–10.7)
Hodgkin lymphoma	3.1	0.0	1.9	0.3	0.2	0.2	10.4 (2.1–29.2)		9.3 (0.5–32.1)
Total	170.9	157.0	165.3	156.7	85.3	120.5	1.1 (0.9–1.3)	1.8 (1.6–2.1)	1.4 (1.2–1.6)

*CI* confidence interval. *The expected rates of cancer death are based on the general population in Korea, adjusted for age and sex.

### Risk factors for PTM

**Figure 1:**
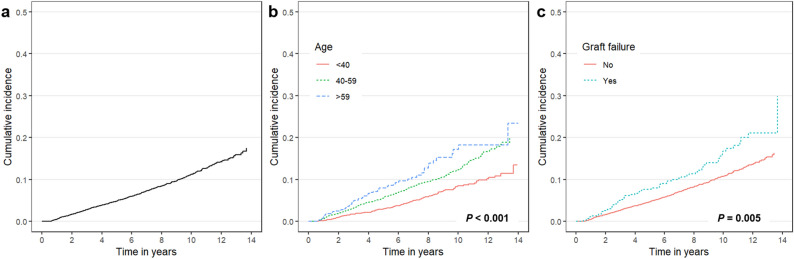
Cumulative incidence rate of post-transplant malignancy after kidney transplantation. (**a**) Total incidence. (**b**) Older age and (**c**) graft failure were associated with post-transplant malignancy.

The risk factors for PTM based on Cox multivariate analysis are shown in Table 5. The risk factors independently related to PTM were older age (40–59 years, hazard ratio [HR] = 1.70; 95% confidence interval [CI] = 1.41–2.06; *P* < 0.001, and over 60 years, HR = 2.30; 95% CI = 1.72–3.06; *P* < 0.001) and graft failure (HR = 1.64; 95% CI = 1.26–2.13; *P* < 0.001). A non-significant relationship between residuals and time was identified (chi-square = 2.30; *P* = 0.682) using the Schoenfeld residuals test to confirm the Cox proportional-hazards assumption (Supplementary Fig. S1). In addition, the Wald chi-square test for goodness of fit was significant (60.98; *P* < 0.001), and variance inflation factors ranged from 1.004 to 1.177 which were less than 10, indicating non-multicollinearity. Figure 1a illustrates the total cumulative incidence of PTM and Fig. 1b,c illustrate the effect of significant factors on cumulative incidence. The estimated cumulative incidence rates of PTM at 1, 2, 5, 10, and 14 years were 0.54%, 1.70%, 4.88%, 11.23%, and 17.49% respectively.

**Table 5:** Univariate and multivariate analysis for post-transplant malignancies.

Variable	Univariate for PTM		Multivariate for PTM*	
	HR (95% CI)	*P*-value	HR (95% CI)	*P*-value
**Age, years**				
< 40	Reference		Reference	
40–59	1.70 (1.41–2.06)	< 0.001	1.70 (1.41–2.06)	< 0.001
> 59	2.29 (1.72–3.05)	< 0.001	2.30 (1.72–3.06)	< 0.001
**Sex**				
Male	Reference		Reference	
Female	1.12 (0.95–1.32)	0.166		
**Induction immunosuppressant**				
Basiliximab	1.15 (0.86–1.56)	0.348		
Anti-thymocyte globulin	1.26 (1.04–1.52)	0.017	1.20 (0.99–1.46)	0.058
**Maintenance immunosuppressant**				
Tacrolimus + MMF + corticosteroid	1.14 (0.96–1.34)	0.135		
Cyclosporine + MMF + corticosteroid	1.03 (0.87–1.23)	0.715		
**Infection**				
CMV disease	1.04 (0.80–1.35)	0.788		
EBV mononucleosis	1.10 (0.15–7.79)	0.928		
Graft failure	1.56 (1.20–2.02)	0.001	1.64 (1.26–2.13)	< 0.001
**Year of transplant**				
2003–2009	Reference		Reference	
2010–2016	1.13 (0.94–1.39)	0.193		

*CMV* cytomegalovirus, *EBV* Epstein–Barr virus, *MMF* mycophenolate mofetil, *HR* hazard ratio, *PTM* post-transplant malignancy. *Variables less than 0.05 of *P*-values in univariate analysis were included in the multivariate analysis.

## Discussion

In the present study, a comprehensive analysis of PTM after kidney transplantation was conducted based on nationwide data from Korea with a lengthy follow-up duration and information on mortality. The SIR of PTM was 3.9, similar to previous studies^11,16,17^. The SMR of total PTM was 1.4, and that of lymphoma showed the highest value (9.3 for Hodgkin lymphoma and 5.5 for NHL). Older age and graft failure were significant risk factors for PTM.

Kidney recipients have higher cases of developing cancer (SIR, 1.7–3.9) compared with the general population^7–12,20–22^. The incidence of PTM varies according to country and type of malignancy. In general, Asian countries (Taiwan, 3.75; Hong Kong, 2.94; and Japan, 2.78)^10,12,21^ have higher SIR than Western countries (Canada, 2.5; United Kingdom, 2.4; and United States, 2.1)^7,9,22^. The difference might stem from the number of included patients, hospital- or population-based designs, and the epoch of transplantation.

Regarding the types of PTM, the most common malignancy was thyroid cancer, followed by colorectal, kidney, stomach, and prostate cancer. The high frequency of these cancers demonstrated previously^16,17^ may result from increased surveillance.

When compared with the general population, the cases of Kaposi sarcoma were the most prominent with an SIR of 192.9 despite a small number of incident cases. An immunocompromised state after kidney transplantation could increase the virus infection that plays an important role in the pathology of Kaposi sarcoma^23^. Further, the lower incidence of Kaposi sarcoma on Korean general population^24^ could have caused the substantially high SIR seen in this study. In terms of kidney cancer having high frequency and SIR, our results were consistent with those of previous studies^21,25^. Although the exact mechanism has not been revealed, malignant transformation of cysts developed during kidney failure, the duration of dialysis, and an immunocompromised state due to nephrotoxic drugs might be the cause of these high kidney cancer values^21,26^.

Regarding mortality associated with PTM, only a few population-based studies have dealt with this subject. According to a recently published Australian and New Zealand population-based cohort study, 20% of cancer deaths were reported, which is slightly higher than our results (14.9%)^19^. The most common cause of cancer death was lung cancer (21%), followed by liver cancer (18%) and NHL (16%). This rank is similar to our results (14.6% for liver cancers, 13.5% for lung, and 12.4% for NHL). Smoking is a well-known risk factor for lung cancer^27^ and hepatitis B virus is one of the established risks of liver cancer^16,28^. In particular, South Korea is an epidemic region for this hepatitis virus. Virus vaccination, prohibitions against smoking, and low dose-computed tomography should be recommended for recipients. In addition, the reduction of spicy food intake, eradication of *Helicobacter pylori*, and screening with gastroscopy for stomach cancer (9.0% of cause of death), which still shows a higher proportion of PTM in Asia^11,12^, should be encouraged.

In terms of SMR, the Australian and New Zealand population-based cohort study reported an SMR of 2.9 for all-site cancers^19^. In the Asian population, a similar SMR value of 2.3 was reported based on the Hong Kong Renal Registry^21^. A population-based cohort study in Canada found an SMR of 2.8; however, the SMR decreased to 1.9 when recipients with cancers before transplantation were excluded^18^. In this study, we presented the SMR for Korea and found a lower SMR (1.4) than the SMRs for Australian and New Zealand, and Hong Kong studies. However, the SMR was still significantly elevated, similar to the previous Canadian study. The exclusion of recipients with pre-transplant malignancies is likely the cause of these results.

The cancer type with the highest SMR was lymphoma (9.3 for Hodgkin lymphoma and 5.5 for NHL). When compared with our results, higher SMR values have been reported in previous studies (42.2 for Australian and New Zealand; 18.2 for Hong Kong; and 14.1 for Italy)^6,19,21^. These different SMR values might be attributed to the higher SIRs of lymphoma in previous studies mostly conducted in Western countries.

Multivariate analysis revealed that the recipient’s age at transplantation and graft failure were significant risk factors for development of PTM. Concordant with our results, the recipient’s age has been widely reported to be a risk factor for PTM^11^. The close relationship between graft failure and development of cancers has also been confirmed in previously reported studies^29^. Immunosuppression associated with graft failure might predispose to the development of PTM. Therefore, alternative regimens to current immunosuppressants such as mammalian target of rapamycin inhibitors that may lead to better outcomes regarding PTM have been investigated^29,30^. In South Korea, most recipients are on a triple therapy regimen that includes a calcineurin inhibitor, mycophenolic acid, and steroids as the major initial maintenance immunosuppressants (81.4%)^31^. Among calcineurin inhibitors, tacrolimus is prescribed far more than cyclosporine, which was reported to be associated with carcinogenic effects^32^. Regarding level, a randomised comparison study demonstrated that recipients with a lower level of cyclosporine had a lower incidence of PTM than those with a normal level^33^. The effects of combination of immunosuppressant, and dosing of them for PTM according to cancer types are still controversial^29^, thus further studies are necessary.

This study had several limitations. The lack of detailed clinical information such as type of pathology, laboratory data, smoking, drinking, donor status, immunologic profiles, family history, dosage of differently combined immunosuppressive drugs, and target drug levels led to restrictions on the analysis of PTM. Moreover, inevitable classification bias could exist because we used registry data based on physicians’ diagnoses. Furthermore, fatal cancers such as early post-transplant lymphoproliferative disorder^34^, which could develop within 6 months after kidney transplantation would be omitted because of predefined criteria based on a previous study investigating de novo non-cutaneous PTM^5^. The precise identification and calculation of SIR and SMR for multiple or recurrent malignancies were also difficult in this type of dataset. Therefore, we considered the first malignancy as PTM due to the strong effect of de novo malignancy on mortality. Further studies focusing on early-onset PTM, multiple and recurrent malignancies, and specific risk factors for PTM are necessary. Hospital-based designs that include participating multi-centres for precise identification of variables and a large study population would complement our limits in the present study. Despite these limitations, the strength of this study is the use of a nationwide population database of kidney recipients with 14 years of lengthy follow-up for late-onset malignancies. Studies handling PTM with SMR using a nationwide data source are rare, particularly in Asia. The relatively large sample size of the entire national population and unbiased measures used in this study could provide reliable information about PTM in kidney recipients.

In conclusion, our study presented a comprehensive analysis of PTM after kidney transplantation, including SIR, SMR, and risk factors. We found that the SIR of malignancy in kidney transplant recipients was 3.9, and provided information about late-onset PTM within 14 years, which might be useful for long-lived recipients. Furthermore, this study is the first to reveal that the SMR of PTM is slightly higher than that in the general population in South Korea. Although increases in cancer incidence of certain types of PTM translate into similar increases in cancer mortality, fatal malignancies significantly related to mortality such as liver and lung cancers, lymphoma, and stomach cancer need more intensive care based on data used in this study. Further, risk factors for PTM including older age and graft failure should be considered when managing transplant recipients. These findings reflecting geographical variation can facilitate the development of cancer prevention strategies and follow-up recommendations for the improvement of the outcome of kidney recipients.

## Methods

### Study design

This was a retrospective and observational cohort study that used prospectively registered national data sets for reimbursement purposes. All patients who underwent kidney transplantation procedures (Z94.0 code of the International Classification of Disease, 10th revision, Clinical Modification [ICD-10-CM]) at any Korean medical centre from January 2003 to December 2016 were included. We included first malignancy after kidney transplantation as PTM because the outcome of multiple or recurrent cancers could be affected by the type of first malignancy. De novo malignancy exhibited a more aggressive tendency, and was one of the leading causes of mortality according to previous studies^35,36^. The recipients with pre-transplant cancers were excluded in order to focus on de novo malignancy^18^. Cancer cases that occurred within 6 months after kidney transplantation were excluded from analysis due to the possibility of undiagnosed malignancies before surgery based on the criteria of a previous study for de novo non-cutaneous PTM^5^. Malignancies detected after graft failure were also excluded. We investigated the incidence, mortality, and risk factors related to PTM.

This study was approved by the independent Institutional Review Board of Kosin University Gospel Hospital (KUGH 2017-12-009). The data acquisition number for the National Health Insurance Sharing Service was REQ0000019170. This study was conducted in accordance with the Declaration of Helsinki. The need for informed consent was waived because anonymity of personal information was maintained.

### Study population (patient selection)

The study included all patients who had been listed for kidney transplantation from January 2003 to December 2016 in the Health Insurance Review and Assessment Service (HIRA). The patients were registered in the HIRA database after kidney transplantation, as defined by the ICD-10-CM code Z94.0. During this period, 18,822 patients were enrolled in the database. We excluded 6089 patients who were not diagnosed with cancer as the main disease, and registered in the reimbursement program for cancer patients properly. Additionally, 1898 patients who were diagnosed with cancer before transplantation, and 430 patients who were diagnosed with cancer within 6 months after kidney transplantation or after graft failure were not included. Patients under 20 years (n = 410) and 53 patients who underwent other organ transplantations were also excluded. Twenty-seven recipients with missing variables such as age, and sex were not included. The final cohort consisted of 9915 patients and included 598 patients with PTM (Supplementary Fig. S2). The records of medical visits, demographic characteristics, and death status were collected from the HIRA database for all kidney recipients with and without PTM.

### Study variables

We collected the following demographic data and baseline characteristics of kidney recipients from the HIRA database: age, sex, immunosuppressive agents, the presence of cytomegalovirus (CMV) disease, Epstein-Barr virus (EBV) mononucleosis, year of transplantation, cancer type, and date of mortality. CMV infection included CMV diseases (mononucleosis, pneumonitis, hepatitis, and pancreatitis) and the post-transplant administration of antiviral agent (ganciclovir or valganciclovir)^37^. The ICD-10-CM codes for CMV disease were B27.1, B25.0, B25.1, B25.8, and B25.9. In terms of EBV status, the ICD-10-CM code B27.0 was used. We defined cases of more than 10 dialysis sessions after 90 days post kidney transplantation as graft failure^38^.

### Data source

The data used in this study were obtained from the HIRA database, which is based on the NHI system operated by the Korean government. Healthcare institutions submit the medical data for all inpatients and outpatients in electronic format to the HIRA for reimbursement purposes. The claims data integrated by HIRA include all healthcare utilisation information on inpatients and outpatients. Data about the demographic characteristics of the patients, principal diagnosis, prescription history, and performed procedures based on ICD-10-CM codes are included in this database. In this study, we obtained all data about kidney recipients from the RID program of the HIRA database who were registered between January 2003 and the end of December 2016. The Korean government assigned kidney transplantation to the RID system to reduce patient payments. To ensure the quality of registration in the RID program, diagnosis must be based on the uniform criteria provided by the NHI and must be reviewed by the corresponding healthcare institution before submission to the NHI, which confirms the medical record of each registered individual. Therefore, the data registered in the RID registry that are linked with the national health insurance system are verified and reliable^39,40^.

Causes of death for the deceased kidney recipients were also analysed by linking the Statistics Korea data with the HIRA database. In the Statistics Korea data, the causes of death are documented according to the ICD-10 codes, which are verified by physicians at the time of death, and information regarding all casualties was included. Cancer incidence and mortality of the general population were also obtained from the Statistics Korea data.

### Statistical analysis

We evaluated the incidence, mortality, and risk factors for PTM. Descriptive statistics were used for patient characteristics associated with PTM. Comparisons of nominal and continuous variables between groups were assessed using the Chi-square and Mann–Whitney *U* tests, respectively. SIR was calculated as the number of observed PTM cases divided by the expected number of malignancies based on the person-years at risk and the cancer incidence rates in the general population. The SMR was calculated as the observed rates divided by the expected rates of mortality in order to compare with the general population^21^. The PTM related mortality was defined as primary cause of death due to PTM^21^, and was analysed based on the data provided by Statistics Korea. Multivariate Cox proportional hazards regression models were used to examine the variables having *P* values less than 0.05, which were considered to be statistically significant based on univariate analysis^41,42^ for PTM.

Statistical analyses were performed using R statistical software (version 3.4.4; R Foundation for Statistical Computing, Vienna, Austria) and SAS statistical analysis software (version 9.4; SAS Institute Inc., Cary, NC, USA). *P* values less than 0.05 were considered statistically significant.

## Supplementary information

Supplementary Information.

## Data Availability

All data generated or analysed during this study are included in this published article (Tables and Figures) and available from the corresponding author on reasonable request. The additional raw data are available on request to the National Health Insurance Service, Korea.
