# Supplementary material for: Incidence of malignancy and related mortality after kidney transplantation: a nationwide, population-based cohort study in Korea
Source: Sci Rep. 2020 Dec 8;10:21398. doi: 10.1038/s41598-020-78283-5 (PMC7722878; doi:10.1038/s41598-020-78283-5)
Supplement: Supplementary file 1 — Supplementary Information. [file 41598_2020_78283_MOESM1_ESM.pdf]

Supplementary Information

Incidence of malignancy and related mortality after kidney transplantation: A nationwide, population-based cohort study in Korea

Seri Jeong[1], Ho Sup Lee[2], Seom Gim Kong[3], Da Jung Kim[2], Sangjin Lee[4], Min-Jeong Park[1], Wonkeun Song[1], John Hoon Rim[5,6] & Hyung Jik Kim[7*]

[1]Department of Laboratory Medicine, Kangnam Sacred Heart Hospital, Hallym University College of Medicine, Seoul, 07441, South Korea. [2]Department of Hematology-Oncology, Kosin University College of Medicine, Busan, 49267, South Korea. [3]Department of Pediatrics, Kosin University College of Medicine, Busan, 49267, South Korea. [4]Graduate School, Department of Statistics, Pusan National University, Busan, 46241, South Korea. [5]Department of Pharmacology, Yonsei University College of Medicine, Seoul, 03722, South Korea. [6]Department of Medicine, Physician-Scientist Program, Yonsei University Graduate School of Medicine, Seoul, 03722, South Korea. [7]Department of Internal Medicine, Hallym University Sacred Heart Hospital, Hallym University College of Medicine, Anyang, 14068, South Korea.

[*] Corresponding author: Hyung Jik Kim

Department of Internal Medicine, Hallym University Sacred Heart Hospital, Hallym

University College of Medicine, 22, Gwanpyeong-ro 170 beon-gil, Dongan-gu, 14068, Anyang-si, Gyeonggi-do, South Korea.

Tel: (+82) 31-380-3720, Fax: (+82) 31-386-2269

E-mail: hyung@hallym.or.kr

Supplemental table and figure legends

Supplementary Figure S1. Plots of scaled Schoenfeld residuals test for proportional hazards assumption.

Supplementary Figure S2. Flow chart of patient selection.

Supplementary Table S1. Standardised incidence ratios of total, and the major types of post-transplant malignancies according to the age classes.

Supplementary Figure S1.

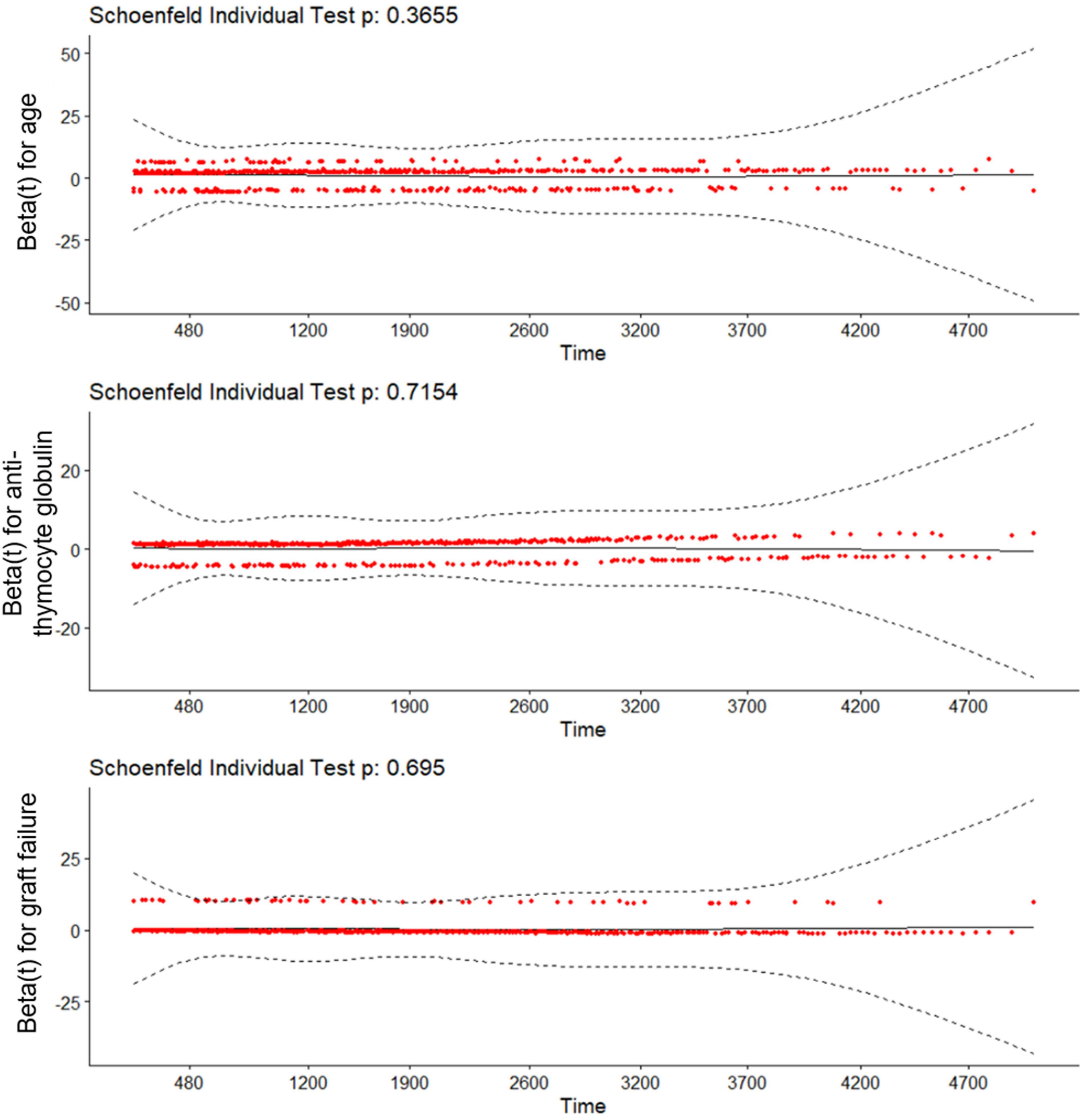

Supplementary Figure S2.

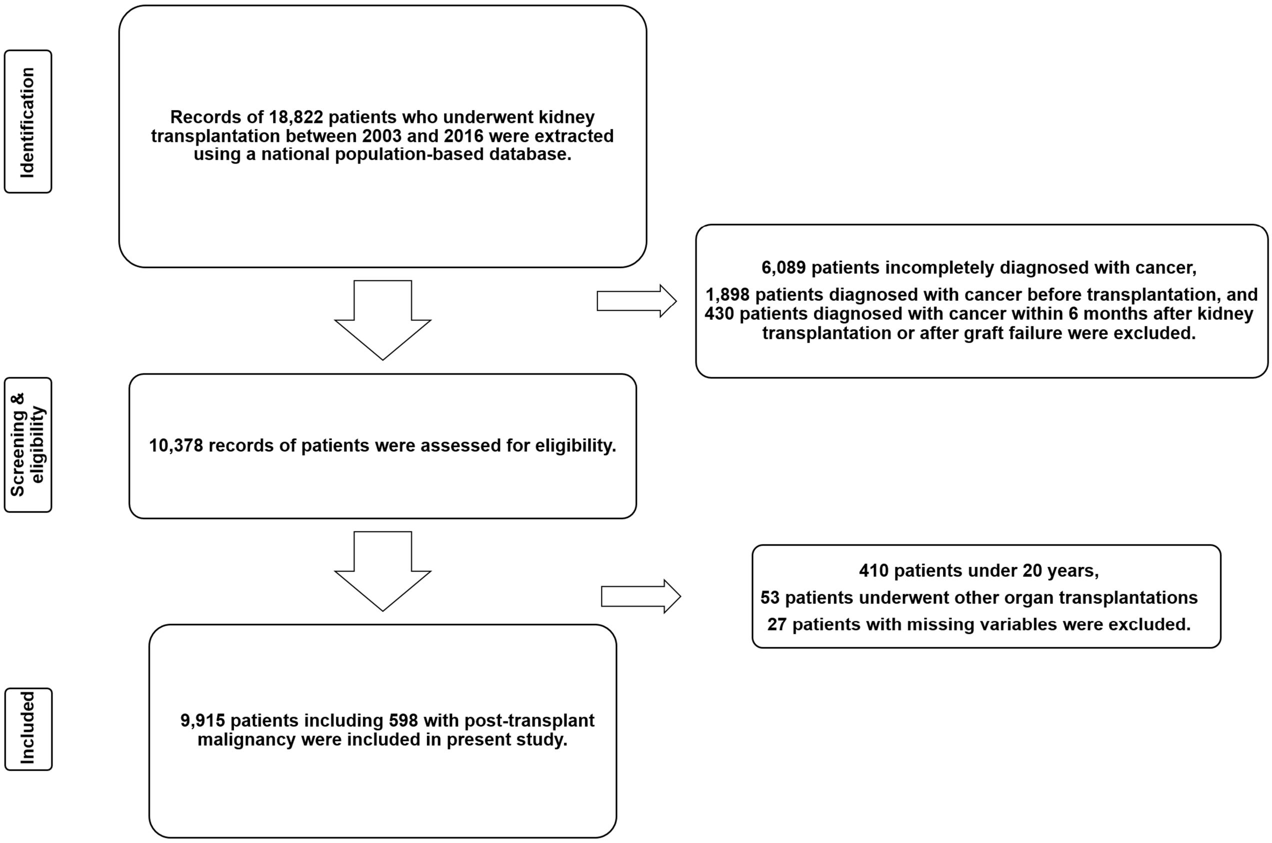

Type of cancer	Observed rates of cancer cases			Expected rates of cancer cases			Standardised incidence ratio (95% CI)		
	Male	Female	Total	Male	Female	Total	Male	Female	Total
Total cancer									
<40	643.1	962.8	769.1	26.7	69.9	47.9	24.0 (22.2-26.0)	13.8 (12.9-14.7)	16.1 (14.9-17.2)
40-59	1242.7	1303.7	1267.7	431.9	496.3	463.2	2.9 (2.7-3.0)	2.6 (2.5-2.8)	2.7 (2.6-2.9)
> 59	1797.4	1400.6	1644.4	2237.8	950.9	1413.8	0.8 (0.8-0.8)	1.5 (1.4-1.6)	1.2 (1.1-1.2)
Thyroid cancer									
<40	66.0	240.7	134.9	7.6	34.7	20.8	8.7 (6.7-11.0)	6.9 (6.1-7.9)	6.5 (5.4-7.7)
40-59	102.6	246.0	161.4	30.8	159.4	94.6	3.3 (2.7-4.0)	1.5 (1.4-1.7)	1.7 (1.4-2.0)
> 59	159.8	382.0	245.4	19.4	63.9	44.0	8.2 (7.0-9.6)	6.0 (5.4-6.6)	5.6 (4.9-6.3)
Colorectal cancer									
<40	74.2	139.4	99.9	2.8	2.3	2.5	26.6 (20.8-33.2)	61.4 (51.5-72.4)	40.0 (32.4-48.4)
40-59	125.4	155.8	137.9	68.9	42.4	55.7	1.8 (1.5-2.2)	3.7 (3.1-4.3)	2.5 (2.1-2.9)
> 59	119.8	191.0	147.3	316.5	163.5	220.5	0.4 (0.3-0.5)	1.2 (1.0-1.3)	0.7 (0.6-0.8)
Kidney cancer									
<40	115.4	88.7	104.9	1.2	0.7	0.9	95.1 (78.2-113.7)	132.4 (106.1-162.7)	110.8 (90.3-133.7)
40-59	159.6	90.2	131.1	14.9	5.7	10.3	10.7 (9.1-12.5)	15.8 (12.7-19.4)	12.7 (10.6-15.1)
> 59	159.8	0.0	98.2	41.9	15.1	25.1	3.8 (3.2-4.4)	0.0	3.9 (3.2-4.8)

Supplementary Table S1. Standardised incidence ratios of total, and major types of post-transplant malignancies according to the age classes.

CI, confidence interval.
